# Fine mapping and functional analysis of the candidate gene *OFP17*, regulating both seed and fruit sizes in cotton

**DOI:** 10.1002/tpg2.70195

**Published:** 2026-01-31

**Authors:** Min Xu, Liuchun Feng, Susu Liu, Zhechang Zhang, Chenhui Zhou, Guoli Feng, Ningshan Wang, Nijiang Ai, Baoliang Zhou

**Affiliations:** ^1^ State Key Laboratory of Crop Genetics & Germplasm Enhancement and Utilization, Collaborative Innovation Center for Modern Crop Production co‐sponsored by Jiangsu Province and Ministry of Education, Cotton Germplasm Enhancement and Application Engineering Research Center (Ministry of Education), College of Agriculture Nanjing Agricultural University Nanjing Jiangsu People's Republic of China; ^2^ Shihezi Agricultural Science Research Institute Shihezi Xinjiang People's Republic of China

## Abstract

Single cotton boll weight (BW) and seed index (SI, 100‐seed weight) are key determinants of lint and seed yield, yet their genetic basis remains poorly understood. Here, a stable interploidy introgression line with small boll from *Gossypium arboreum* in *Gossypium hirsutum*, NB298, was employed to map quantitative trait loci (QTLs) of BW and SI. A moderate/minor‐effect QTL on ChrA05 was preliminary mapped in the F_2_ population of NB298 and TM‐1, with the phenotypic variation explained of 6.21% and 1.65% for BW and SI, respectively, named *q(BW+SI)‐A05*. Sixteen simple sequence repeat and insertion and deletion (InDel) markers were further developed for genotyping of individuals in F_2_ and F_3_ populations. A total of seven QTLs for three yield‐related traits (BW, SI, and boll number per plant) were detected. Among them, stable QTLs for BW and SI were co‐located in the interval between InDel‐4353 and InDel‐4370 in both F_2_ and F_3_ populations, with a length of 1354.55 kb. Then, the substitution mapping method demonstrated the QTL was located in an interval of 468.155 kb between the markers of InDel‐4365 and InDel‐4373. Comparisons of BW and SI between introgression lines with/without the introgression fragment showed they were significantly different. Combined with the RNA‐seq of the ovules of 0, 3, and 5 days post anthesis, three differentially expressed genes were obtained and subjected to virus‐induced gene silencing and overexpressing in cotton and ectopic expression in *Arabidopsis thaliana*. The identification of *OFP17* (where OFP is ovate family protein) provides a molecular target for manipulating seed and fruit size to enhance cotton yield.

## Core Ideas

- A boll weight and seed index related quantitative trait locus (QTL), *q(BW+SI)‐A05*, was fine‐mapped to an interval of 468.155 kb.
- By the combination of RNA‐seq, virus‐induced gene silencing or overexpression in cotton, and ectopic expression in *Arabidopsis*, *OFP17* (where OFP is Ovate family protein) was considered as a candidate gene.
- The *OFP17* expression level influences ovule growth and development, as well as seed and fruit development.

## Plain Language Summary

Cotton yield depends on traits such as boll weight (BW, lint and seed weight within a boll) and seed index (SI, 100‐seed weight), which were improved during domestication. These traits are complex and influenced by many genes. BW as one of the three yield component factors, improving BW is an effective way to increase yield when the total number cotton boll per unit area keeps stable. However, which genetic mechanism behind BW and SI remains unclear. In this study, a cross between *Gossypium arboreum* and *Gossypium hirsutum* was used to identify a genetic region controlling both seed and fruit size. The gene *OFP17* (where OFP is ovate family protein) was found to negatively regulate these traits. This finding helps explain the genetic control of seed and fruit size and could support cotton yield improvement.

**Abbreviations**
BR: Brassinosteroids
BW: boll weight
CDS: coding DNA sequence
DEGs: differentially expressed genes
IL: introgression line
InDel: insertion and deletion
LI: lint index
LOD: logarithm of odds
LP: lint percentage
OE: overexpression line
OFP: ovate family protein
PC: principal component
PCR: polymerase chain reaction
QTL: quantitative trait locus
SI: seed index
SNP: single nucleotide polymorphism
SSR: simple sequence repeat
VIGS: virus‐induced gene silencing
WGCNA: weighted gene co‐expression network analysis

## 1 INTRODUCTION

Cotton (*Gossypium* spp.) is the world's most important natural textile fiber, and its seed is an important source of plant protein and edible oil. Cotton yield is typically affected by several complex quantitative traits, including boll number per plant (BN), lint percentage (LP) (Frary et al., 2000), single boll weight (BW, lint and seed weight within a boll), seed index (SI, 100‐seed weight), and lint index (LI, lint weight of 100‐seed‐cotton) (Qin et al., 2008), which have been positively selected in cultivated cotton through domestication (L. Fan et al., 2018; Fang et al., 2017; Feng et al., 2019; Gu et al., 2020). BW is one of the three yield component factors, and improving BW is an effective way to increase yield when the total number of cotton bolls per unit area remains stable. BW consists of the weight of seeds and lint fiber within a single boll. SI, the weight of 100 cotton seeds, represents the seed size.

With the cotton genome sequence releasing, the number of yield‐related quantitative trait loci (QTLs) discovered is rapidly increasing by genome‐wide association study or linkage mapping. Numerous studies indicate that QTLs for BW and SI are distributed on almost all chromosomes in cotton genome (Y. Hu et al., 2019; Huang et al., 2020; Li et al., 2015; M. Wang et al., 2019; T. Zhang et al., 2015).

Seed is one of the most important organs for plant propagation. Cotton seed size or SI also has an impact on LP, generating an indirectly effect on cotton yield. Seed size can be described by three main dimensions: length, width, and thickness (Xing & Zhang, 2010). Numerous genes related to seed shapes and sizes are discovered in *Arabidopsis* (Cheng et al., 2014; Garcia et al., 2005; J. W. Wang et al., 2008; Y. Zhang et al., 2020), *Oryza sativa* (rice) (C. Fan et al., 2006; J. Hu et al., 2015; Song et al., 2007; Weng et al., 2008; W. Yang et al., 2021; X. Zhang et al., 2012), and *Glycine max* (soybean) (Duan et al., 2022; Ge et al., 2016; Nguyen et al., 2021; Tang et al., 2017), while in cotton only a handful of genes are considered to be related to seed or organ size. For example, Ahmed et al. (2020) detected a putative BW gene, *GhBRH1_A12*, regulating Brassinosteroids (BR) homeostasis. Due to an insertion and deletion (InDel) at the downstream of the gene, the BR signal cascade in cotton was disrupted, leading to BR insensitivity that impaired cotton development. X. Liu, Hou et al. (2022) found that *GhSI7*, encoding the transcriptional regulator STERILE APETALA, was related to SI and organ size in cotton plants. Feng et al. (2022) fine‐mapped a QTL *q(BW+SI)‐A12‐1* and detected *TIP41L* gene related to BW and SI. P. Wang et al. (2022) found that *GhUBC2L* co‐regulates histone monoubiquitination with *GhUbox8*, regulating gene expression involved in organ development and cell cycle, thereby controlling cotton organ size. While prior work identified regulators of brassinosteroid and transcriptional control pathways, the direct transcriptional repressors involved in organ size modulation remain uncharacterized.

Despite substantial progress in QTL identification from two cultivated tetraploid cotton (*Gossypium hirsutum* and *Gossypium barbadense*) (Ahmed et al., 2020), very few QTLs for BW and SI from diploid cotton such as *Gossypium arboreum* are mapped and cloned, and the molecular mechanisms underlying seed and fruit size regulation in cotton remain unclear. In our recent study, a stable introgression line (IL), NB298, was developed from a cross of *G. hirsutum* acc. TM‐1 (2*n* = AADD = 52) and *G. arboreum* acc. Shixiya 1 (2*n* = AA = 26), being significantly different from the recurrent parent (TM‐1) in BW, SI, and BN (Feng et al., 2021). This study aimed to fine‐map QTLs for BW and SI of *G. arboreum* in *G. hirsutum*, identify candidate genes, and validate their roles through expression analysis and functional assays.

## 2 MATERIALS AND METHODS

### 2.1 Plant materials

In our previous study, chromosome segment ILs were developed to broaden the upland cotton genetic basis using an interploidy cross between *G. hirsutum* acc. TM‐1 (2*n *= AADD = 52) and *G. arboreum* acc. Shixiya 1 (2*n *= AA = 26) (Feng et al., 2021). In the IL population, a stable IL, NB298, significantly differs from the recurrent parent TM‐1 in BW, SI, boll length, and boll width. To fine‐map the QTLs related to the above traits, NB298 was crossed with TM‐1 and produced the secondary segregation populations of F_2_, F_3_, and F_3:4_. A total of 1695 individuals of F_2_ grew in Shangqiu, Henan Province, in 2019. Then 169 F_2_ individuals, which were heterozygous in the putative QTL interval of INTR0048–INTR0049 on ChrA05, were selected for self‐crossing to generate the F_3_ population. A total of 1907 F_3_ individuals were produced and grown in Shangqiu, Henan Province, in 2020. The recombinants in F_3_ generation are picked and grown in Shangqiu in 2021 as F_3:4_ population. A randomized block test was designed in all generations, and cotton was cultivated under the normal conditions at the same location. All the F_3_ and F_3:4_ individuals were further genotyped for QTL mapping with the 16 newly developed markers of the above interval. Both TM‐1 and NB298 were used as controls with three replicates under all environments. The recombinants in the F_3:4_ population were performed twice. Fifteen bolls from each individual plant of F_2_ and F_3_ and 25 bolls from parent plants were harvested to weigh BW and SI. All phenotypic data were calculated and analyzed using Microsoft Excel and SPSS v25.0 (SPSS).

### 2.2 DNA extraction and molecular marker analysis

Total genomic DNA was extracted from young leaves of F_2_/F_3_/F_3:4_ individuals and their parents using a modified cetyltrimethylammonium bromide (CTAB) method (Paterson et al., 1993). The primers for simple sequence repeat (SSR) and InDel markers were designed based on the TM‐1 genome (Y. Hu et al., 2019) and the Shixiya 1 genome (Huang et al., 2020). The markers that showed polymorphism among the A_t_, D_t_, and A_2_ genomes were employed for further genotyping the individuals of the F_2_, F_3_, and F_3:4_ populations. Their amplicons should be different by at least 10 bp for clear detection on gels. Totally, 16 markers were developed for identifying recombinants in the target QTL region. Collinearity analysis for A_2_‐genome (Shixiya 1 genome) and A_t_‐genome (TM‐1 genome) was performed using MCScanX software (Y. Wang et al., 2012).

**Core Ideas**

- A boll weight and seed index related quantitative trait locus (QTL), *q(BW+SI)‐A05*, was fine‐mapped to an interval of 468.155 kb.
- By the combination of RNA‐seq, virus‐induced gene silencing or overexpression in cotton, and ectopic expression in *Arabidopsis*, *OFP17* (where OFP is Ovate family protein) was considered as a candidate gene.
- The *OFP17* expression level influences ovule growth and development, as well as seed and fruit development.

### 2.3 QTL analysis and fine mapping of *q(BW+SI)‐A05*

The JoinMap software (Ooijen & Voorrips, 2001) was used to construct a linkage map from the F_2_ and F_3_ populations, and recombination frequencies were converted into map distances (cM) using the Kosambi mapping function. The resulting linkage map was drawn using the MapChart ver. 2.2 software (Voorrips, 2002). QTLs were detected with WinQTL Cartographer V2.5 software (S. Wang et al., 2005) by composite interval mapping method. Likelihood ratio threshold values (*α* = 0.05) for declaring the presence of QTLs were estimated after 1000 permutations (Doerge & Churchill, 1996). QTLs were searched with a 10‐cM testing window, 1‐cM walking speed, and other default parameters. Logarithm of odds (LOD) value ≥ 3.0 indicates a strong genetic linkage between the locus and trait. QTLs were defined by one‐LOD likelihood intervals on either side of the peak position. The precise position was determined by comparing the phenotypic averages between the recombinants identified in the F_3_ and F_3:4_ populations. The significance of the difference was analyzed by SPSS v25.0 (SPSS).

### 2.4 Preprocessing of RNA‐seq data

Total RNA extraction, library construction, and 150‐bp PE Illumina sequencing were performed as described by Hovav et al. (2015). Eighteen libraries from the ovules of 0, 3, and 5 days post‐anthesis (dpa) in TM‐1 (TM) and NB298 (NB) were prepared, and raw RNA reads were obtained by an Illumina NovaSeq 6000 sequencer. Quality filtering and reads trimming were performed as described in https://www.jianshu.com/p/b8276812c27b by FastQC (Andrews, 2014) and Trimmomatic (Bolger et al., 2014) software. The clean reads were then mapped to the reference genomic sequence (TM‐1 V2.1, Y. Hu et al., 2019) using the Hisat2 tool (Kim et al., 2015), and the counts of the mapped reads were calculated using the HTSeq software with default parameters (Anders et al., 2015).

### 2.5 Differentially expressed genes and weighted gene co‐expression network analysis

The expression level of each gene was normalized using transcripts per million values. The DESeq2 package in the R software was used to analyze differentially expressed genes (DEGs) between samples (Zhao et al., 2021). The distribution of *p*‐values was controlled by Benjamini–Hochberg method at *α* = 0.05 false discovery rate (*q*‐value). The DEGs were detected according to the parameters: | log2 fold change | ≥ 1 and *q*‐value ≤ 0.05. The pattern of gene co‐expression across genotypes and treatments, as well as the relationship between synergistically changed gene sets and phenotypes, was identified using the weighted gene co‐expression network analysis (WGCNA) package in R (Langfelder & Horvath, 2008). To construct the gene co‐expression network based on the appropriate scale‐free topology criterion, set the soft‐thresholding power of *β* = 7, MinModuleSize = 30, and mergeCutHeight = 0.75. Calculate the correlation coefficients between the eigengenes of the modules and the samples or sample treatments to further explore biologically significant modules. Visualization was performed using the Cytoscape software (Shannon et al., 2003).

### 2.6 Virus‐induced gene silencing procedures

Cotton seeds were sown in paper cups filled with peat soil:vermiculite (1:1) and cultured in the greenhouse (16‐h photoperiod; 25°C/23°C day/night temperature; 70% relative humidity). Uniformly growing cotton seedlings were selected for virus‐induced gene silencing (VIGS) test. VIGS experiments were performed as described by Burch‐Smith et al. (2004) and optimized by Gao et al. (2011) and Pang et al. (2013) for cotton. The candidate gene fragments were cloned into the pTRV2 vector for construction of silencing vectors. The primer information used to clone fragments of genes for VIGS is listed in Table S1. In addition, the empty vector TRV:*00* was set as a negative control and TRV:*Gh_CLA* as a positive control to show if the silencing system worked. *Agrobacterium tumefaciens* (GV3101) carrying the silencing vector was resuspended in buffer (10 mM MgCl_2_, 10 mM MES, and 200 µM AS) to an OD_600_ of 1.0 and injected into cotyledon of cotton seedlings (10 days old). The silencing efficiency was further determined by real‐time quantitative polymerase chain reaction (RT‐qPCR). When growing to the three‐leaf stage (15 days post‐infiltration), the leaf length and plant height of the seedlings were observed and evaluated. In parallel, sampled for RNA extraction, respectively.

### 2.7 Overexpression of candidate genes in *Arabidopsis thaliana*

The coding region of the candidate genes were inserted into pBI121‐GUS for construction of overexpression vectors. The primer information used to clone full coding DNA sequence (CDS) of genes is listed in Table S1. Then, the overexpression vectors were transformed into *Arabidopsis thaliana* (Columbia, Col‐0) according to the flower dip method (X. Zhang et al., 2006). *Arabidopsis* seedlings were cultured in a growth chamber during daytime (16 h and 22°C) and nighttime (8 h and 18°C). Until the T_2_ generation, >10 transgenic lines (overexpression line [OE]) of each gene and three transgenic homozygous lines were randomly selected to compare seed sizes. The T_2_ seeds were photographed by magnifying 90‐fold with a stereoscope, and 50 seeds were randomly selected and measured by ImageJ software (Schneider et al., 2012), the area of these seeds can represent the size of these seeds.

### 2.8 Quantitative experiments of relative expression

Total RNAs from cotton and Arabidopsis were extracted using the MolPure Plant RNA Kit (YEASEN) according to the manufacturer's instructions. When the qualified RNA was obtained, cDNA was synthesized using the Hifair III 1st Strand cDNA Synthesis Kit (YEASEN). Following that, the cDNA was used for RT‐qPCR using the Hieff qPCR SYBR Green Master Mix (YEASEN) and LightCycler 480 (Roche Diagnostics GmbH) instruments. *Histone 3* in cotton and *UBQ5* in Arabidopsis were used as internal controls, respectively. The primer information is listed in Table S2. Each experiment was performed with three biological replicates. The 2^−ΔΔCT^ method was used to calculate the relative expression level (Livak & Schmittgen, 2001). Values are expressed as means ± standard deviation (SD).

## 3 RESULTS

### 3.1 Phenotypic performance of NB298 and its introgression segments

**FIGURE 1:**
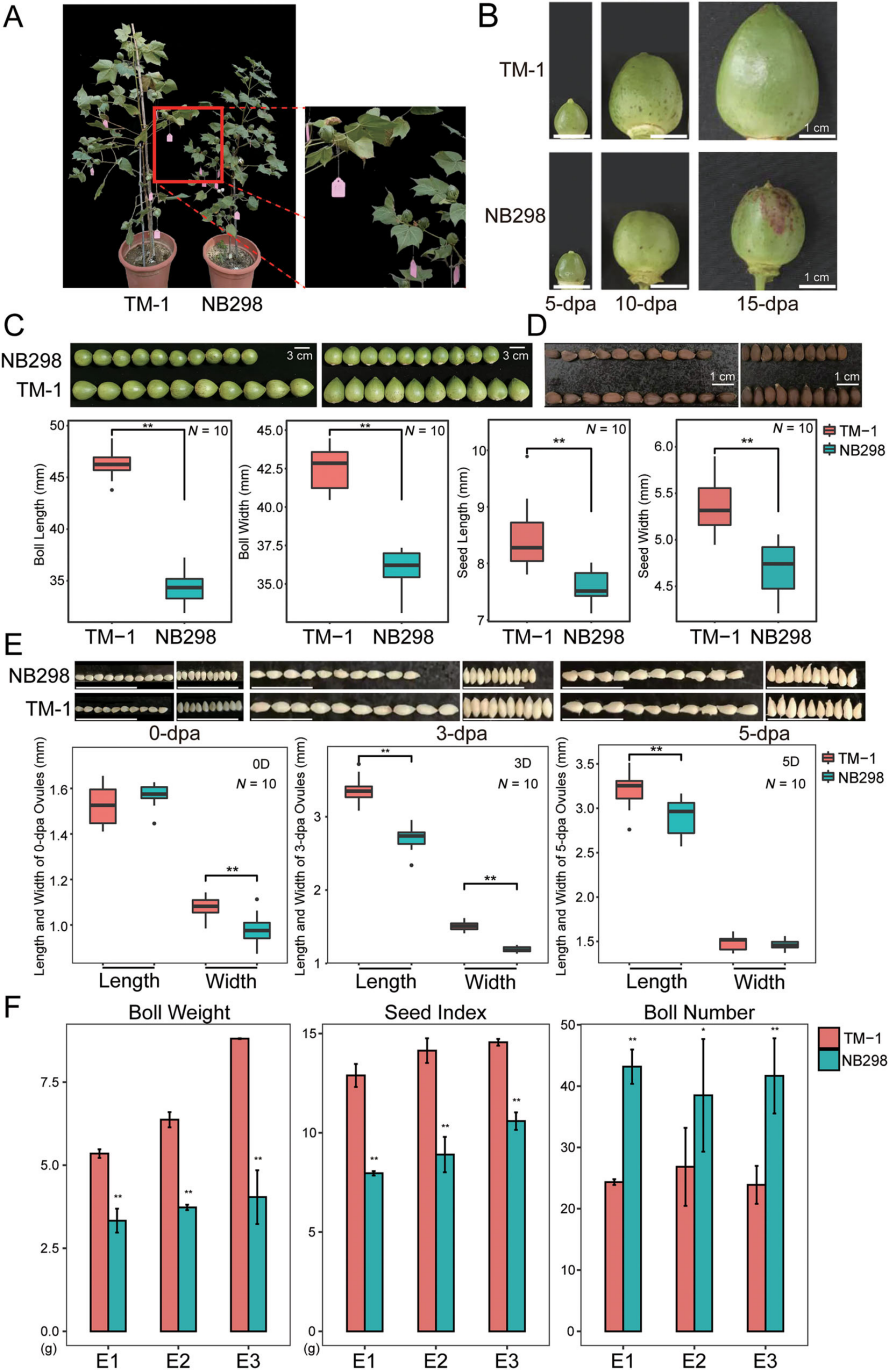
Morphological traits of NB298 and TM‐1. (A) Plant shapes of TM‐1 and NB298; (B) boll shapes measured on 5, 10, and 15 dpa in TM‐1 and NB298, scale bar = 1 cm; (C) boll sizes of NB298 and TM‐1 on 30 dpa, scale bar = 3 cm; (D) ovule sizes on 0, 3, and 5 dpa in NB298 and TM‐1, scale bar = 1 cm; (E) seed sizes of NB298 and TM‐1, scale bar = 1 cm; and (F) comparison of boll weight, seed index, and boll number between TM‐1 and NB298 in three environments. E1, Dangtu, Anhui province in 2018; E2, Shangqiu, Henan province in 2019; E3, Shangqiu, Henan province in 2020, * and ** indicates the significance at the 0.05 and 0.01 level, respectively.

In our previous study, Feng et al. (2021) developed a set of 289 ILs from *G. arboreum* in the *G. hirsutum* background and further evaluated their yield‐related traits in five environments. It was found that the IL, NB298, showed a relatively small phenotype (Figure 1A) with small bolls (Figure 1B,C), small seeds (Figure 1D), and small ovules (Figure 1E). The yield‐related traits fluctuated across environments, but NB298 always showed lower BW (3.33 ± 0.36–4.04 ± 0.81 g vs. 5.35 ± 0.13–6.81 ± 0.01 g) and SI (7.96 ± 0.11–10.59 ± 0.44 g vs. 12.89 ± 0.58–14.56 ± 0.17 g) and more BN (43.17 ± 2.8–41.67 ± 6.13 vs. 24.34 ± 0.47–23.89 ± 3.1) than TM‐1 (Figure 1F; Table S3).

**FIGURE 2:**
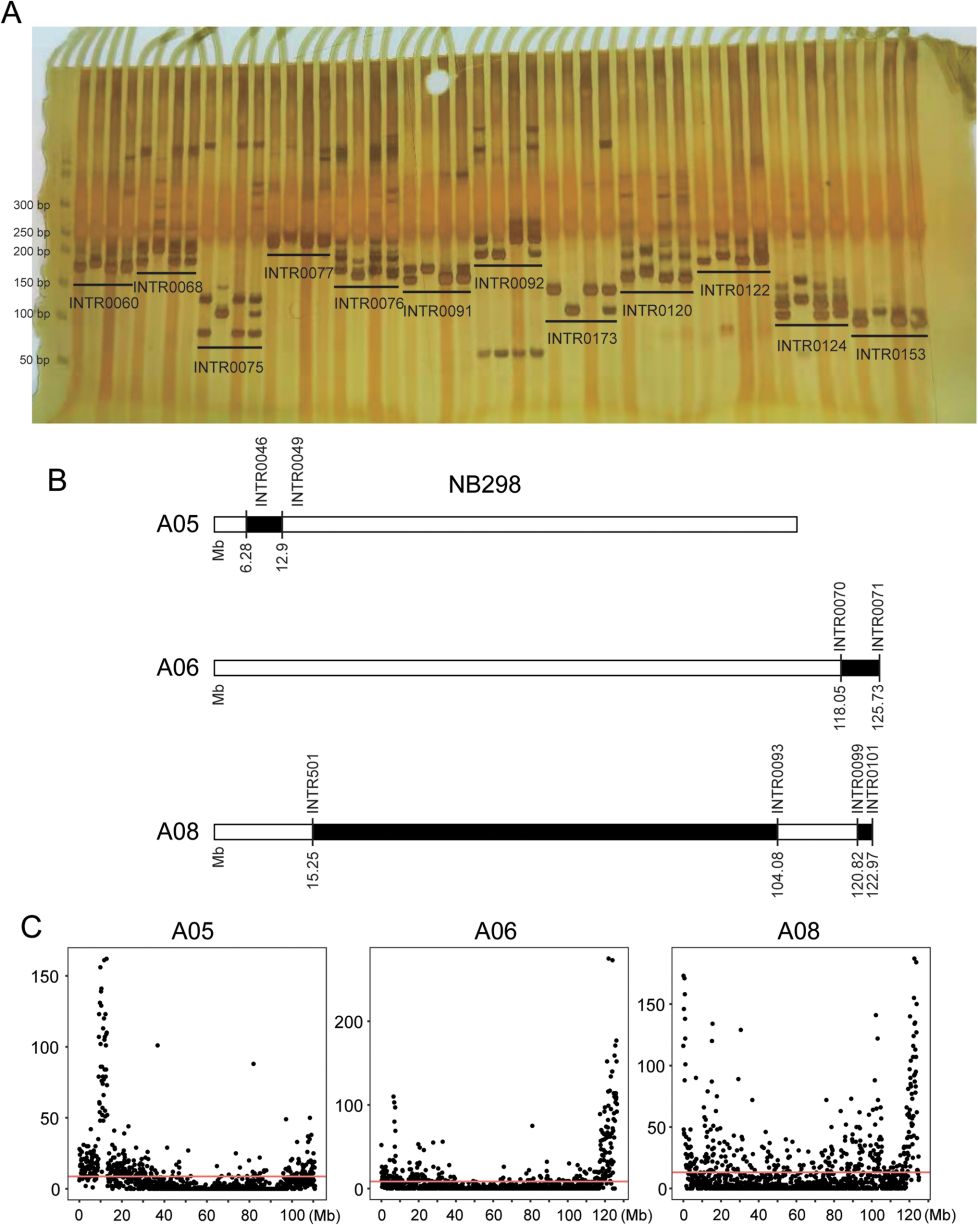
Distribution of introgression segments in NB298. (A) Partial electrophoresis results of polymerase chain reaction (PCR) amplicons between parents and NB298. The primer names of the markers are below the black line. Each four lanes represents NB298, Shixiya 1 (A_2_A_2_), TM‐1 (AADD), and synthetic amphiploid between *Gossypium hirsutum* × *Gossypium arboreum* (AADDA_2_A_2_), respectively. (B) White regions represent the *G. hirsutum* background, black regions represent the introgression segments from *G. arboreum*, the both ends of the introgression fragment are the physical locations of the markers; (C) single nucleotide polymorphism (SNP) distribution on chromosomes A05, A06, and A08 was analyzed using RNA‐seq data. The horizontal axis indicates the positions on chromosomes and the vertical axis shows the number of variations (SNPs or insertion and deletion [InDel]). 100 Kb size sliding window and 10 Kb step size were employed for the number of variations within the window.

A set of markers in our laboratory for identifying exogenous fragments of *G. arboreum* was used to identify NB298 (Figure 2A). There are 47 polymorphic SSR or InDels markers (Table S4) in introgression fragments, with five markers on ChrA05, eight markers on ChrA06, and 34 markers on ChrA08. It was found that NB298 has four introgression fragments from three chromosomes of *G. arboreum*, namely, three small fragments on ChrA05 (6.62 Mb), ChrA06 (7.68 Mb), and ChrA08 (2.15 Mb), as well as a large fragment (88.83 Mb) on ChrA08 (Figure 2B).

Furthermore, RNA‐seq data were used to analyze single nucleotide polymorphisms (SNPs) between TM‐1 and NB298 and identify introgression fragments. The results demonstrated that the intervals with >50 SNPs within 100 Kb were A05 (9.2–12.9 Mb), A06 (117.3–126.4 Mb), and A08 (15.1–103.1 Mb and 119.3–123.8 Mb) (Figure 2C), being consistent with the results identified by the genomic marker.

Collinearity refers to the distribution or arrangement relationship of homologous genes within a species or between different species. Collinearity analysis of the A_2_ and A_t_ genomes can intuitively reveal the evolutionary relationship between these two genomes (Figure S1A). There are several inversions in the introgression fragment on Chr08 (Figure S1B), which may reduce the recombination of the introgression fragment in the segregating population.

### 3.2 QTL analysis of three yield‐related traits in F_2_ and F_3_ populations

Three secondary segregation populations of NB298 and TM‐1, namely, F_2_, F_3_, and F_3:4_ populations, were constructed for QTL mapping of BW, SI, and BN. The average values of the three yield‐related traits in the F_2_ and F_3_ populations were close to those of TM‐1, and the absolute values of skewness were ˂1. Combined with the frequency distribution diagrams of these three phenotypes, it is considered that these three phenotypes conform to a normal distribution and are suitable for QTL mapping research (Figure S2; Table S5). SI showed a low coefficient of variation (12.91% in F_2_ and 13.54% in F_3_), while BW (18.92% and 18.14%) and BN (39.45% and 52.50%) were much higher (Table S5). The correlation analysis showed that significant correlations were observed among the above traits (Table S6). The significant positive correlations existed between SI and BW, BN and BW, or BN and SI.

The initial genotyping of F_2_ populations was carried out using 47 polymorphic SSRs or InDels markers. JoinMap software was employed to construct a genetic linkage map. Three linkage groups, namely, chromosomes A05, A06, and A08, were constructed (Figure S3A). Two QTLs for two traits (BW and SI) were detected on ChrA05 in the F_2_ population (Table S7; Figure S3B). The INTR0048–INTR0049 interval (3517.76 kb) showed large effect on BW and SI. More importantly, *qBW‐A05*, near the INTR0048 marker, was also reported by Feng et al. (2021) in four environments. Therefore, INTR0048–INTR0049 interval was considered as a stable candidate QTL interval for further fine‐mapping, named as *q(BW+SI)‐A05*.

To map *q(BW+SI)‐A05* into a narrow region, we further developed 16 InDel markers to increase marker density (Figure S3C). Seven QTLs for three yield‐related traits were detected on ChrA05 in the F_2_ and F_3_ populations (Table S8; Figure S3D,E). *qBW‐A05‐1* and *qBW‐A05‐2* were located on the same overlapped interval of InDel‐4353–InDel‐4370; and *qSI‐A05‐1* and *qSI‐A05‐2* were also on the same overlapped interval of InDel‐4353–InDel‐4370 (1354.55 kb). Therefore, the InDel‐4353–InDel‐4370 interval is very important for BW and SI. And the recombinants associated with this interval were selected for further fine mapping.

### 3.3 Fine mapping of *q(BW+SI)‐A05* by the substitution mapping method

**FIGURE 3:**
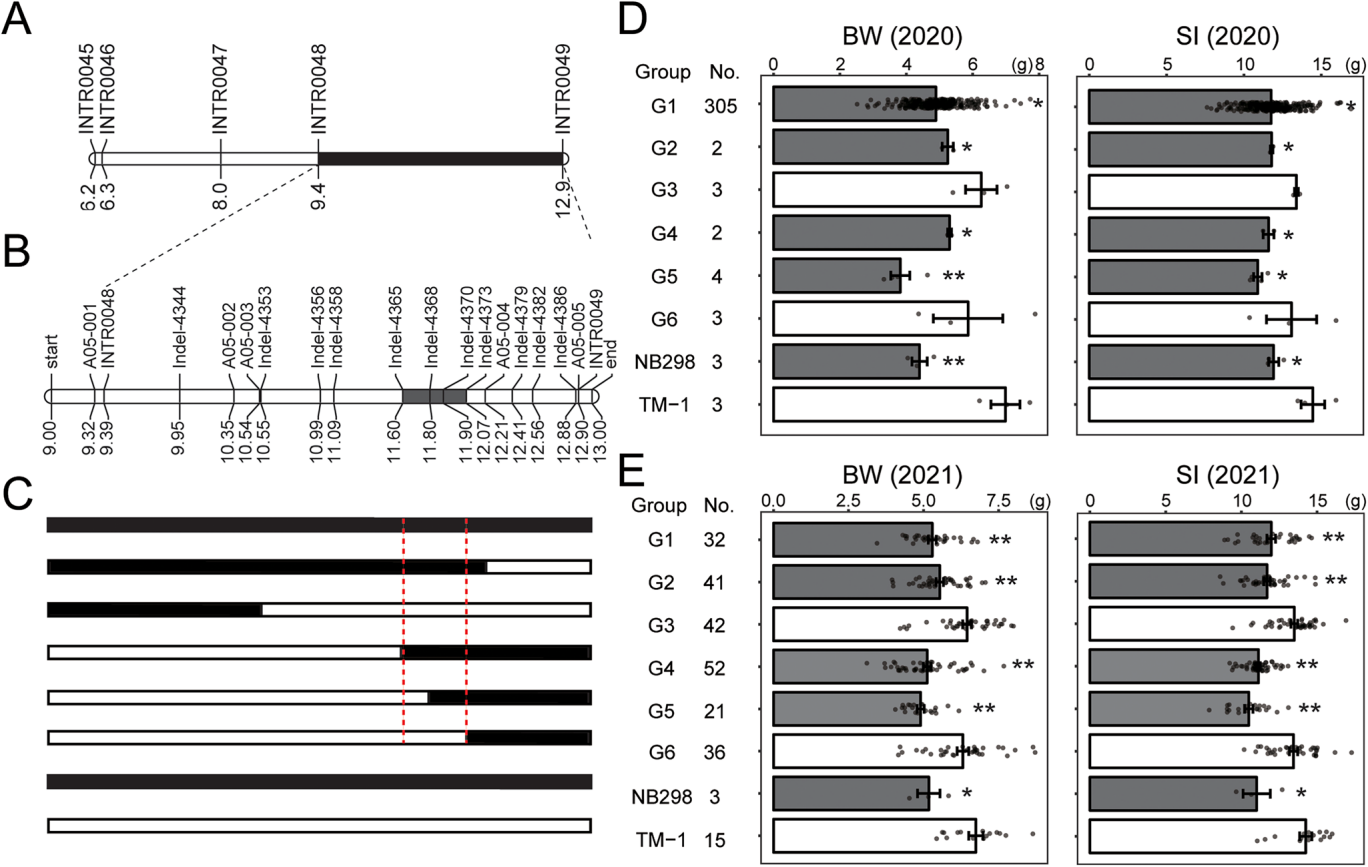
*q(BW+SI)‐A05* was fine‐mapped by the substitution mapping method with recombinants in two environments. (A) Physical map of the introgression segment on ChrA05; (B) the physical location of the newly developed markers in the *q(BW+SI)‐A05* interval; (C) introgression segments in the recombinants; (D) BW and SI of the homologous recombinants derived from the F_3_ population in 2020; and (E) BW and SI of the recombinants in F_3:4_ population derived from the homologous F_3_ individuals in 2021. The white and black bars represent the genotypes of TM‐1 and NB298, respectively. The *q(BW+SI)‐A05* was mapped into a 433.013 kb interval indicated by red dashed lines, and the molecular markers on both sides were insertion and deletion (InDel)‐4365 and InDel‐4373. * and ** indicates the significance at the 0.05 and 0.01 levels, respectively.

We selected the recombinants in the target regions of INTR0048∼INTR0049 for substitution mapping. Finally, 319 recombinants in F_3_ were detected and grouped into six classes (G1–G6) based on their breakpoints (Table S9). These groups were further selected and grown in Shangqiu, Henan Province, China, in 2021 as F_3:4_ populations. Comparisons were performed among groups in F_3_ (2020) and F_3:4_ (2021) for BW and SI by student's *t*‐test, respectively (Figure 3). The results indicated BW and SI in G1, G2, G4, and G5 were significantly lower than that in TM‐1, but they were not significantly different from that in NB298. And BW and SI in G3 and G6 were significantly higher than that in NB298, but they were not significantly different from that in TM‐1 (Table S9). Taken together, *q(BW+SI)‐A05* was supposed to be located in the InDel‐4365–InDel‐4373 interval of 468.155 kb in *G. hirsutum* or 433.013 kb in *G. arboreum*.

### 3.4 DEGs in ovules of NB298 and TM‐1 by RNA‐seq

A total of 18 cDNA libraries of ovules (0, 3, and 5 dpa) from NB298 and TM‐1, respectively, were sequenced to explore genes associated with BW and SI. The GC (guanine and cytosine) content for 18 samples ranged from 41% to 43%, and the Q30 value was 92.07%–93.91%. Clean reads were obtained, ranging from 15.40 to 27.85 million, with 95.56%–97.11% of overall alignment rate and 75.47%–79.41% of unique alignment rates to TM‐1 genome (V2.1) (Y. Hu et al., 2019) with Hisat2 software, and the average sequencing depth ranged from 16.12× to 35.27×. The specific information of each sample is provided in Table S10. Principal component analysis was performed on the differences in developmental ovules from NB298 and TM‐1. The most significant principal component (PC1) explained 63% of the differences among 0, 3, and 5 dpa ovules. The PC2 accounted for 17% of the variation between NB298 and TM‐1 (Figure S4A). Between TM‐1 and NB298, there are 647 DEGs in 0 dpa ovules, 1639 DEGs in 3 dpa ovules, and 1172 DEGs in 5 dpa ovules (Figure S4B). Note that 378 DEGs were shared in all three periods, and 2399 DEGs in the union of the three periods were used for further analysis (Figure S4C).

### 3.5 Identification of hub genes controlling BW/SI by gene co‐expression network analysis of 2399 DEGs

WGCNA analysis was used to describe the association of the differential gene co‐expression modules with ovule growth and development. Gene expression networks were constructed for 2399 DEGs of ovules at different developmental stages by soft threshold of 7 based on a scale‐free power law distribution (Figure S5A,B). Gene module associations with treatment were analyzed by calculating Pearson correlation coefficients. A total of nine modules were identified in the dendrogram (Figure S5C,D). As correlation coefficients were 0.99 and −0.95 (Figure S5E) and the gene significance was 0.75 and 0.70 (Figure S6A), we focused on the two modules (blue and turquoise) (Figure S6): the genes in the blue module being highly expressed in the NB298 ovule, whereas genes in the turquoise module being highly expressed in the TM‐1 ovule. Genes with the highest degree of connectivity in essential modules are known as hub genes, implying they may have vital biological functions in controlling traits. Therefore, the size differences in ovules/fruits (0, 3, and 5 dpa) between TM‐1 and NB298 could be associated with the hub genes in the blue and turquoise modules.

### 3.6 Functional analysis of putative genes in cotton plants by VIGS

By the combination of fine‐mapping and WGCNA analysis of RNA‐seq, 11 candidate genes from blue module (*GH_A05G1302*, *GH_A05G1367*, *GH_A06G2242*, *GH_A08G0993*, *GH_A08G2485*, and *GH_A08G2493*) and turquoise module (*GH_A05G1324*, *GH_A06G1903*, *GH_A08G1638*, *GH_A08G2341*, and *GH_A08G2821*) were selected for functional analysis by VIGS (Table 1).

**TABLE 1:** Candidate hub genes in the introgression region.

Gene ID in *Gossypium hirsutum*	Gene ID in *Gossypium arboreum*	Gene annotation	Module	QTL
*GH_A05G1302*	*Gar05G13380*	Ovate family proteins 17	Blue	*q(BW+SI)‐A05*
*GH_A05G1324*	*Gar05G13620*	Cytochrome c oxidase assembly protein 19	Turquoise	*q(BW+SI)‐A05*
*GH_A05G1367*	*Gar05G14070*	Transmembrane 9 superfamily member 7	Blue	*q(BW+SI)‐A05*
*GH_A06G1903*	*Gar06G22750*	NA	Turquoise	
*GH_A06G2242*	*Gar06G26100*	Pyruvate dehydrogenase E1 component subunit beta‐2	Blue	
*GH_A08G0993*	*Gar08G10510*	40S ribosomal protein S11	Blue	
*GH_A08G1638*	*Gar08G18470*	GATA transcription factor 4	Turquoise	
*GH_A08G2341*	*Gar08G26550*	Eukaryotic translation initiation factor 3 subunit A	Turquoise	
*GH_A08G2485*	*Gar08G28180*	Protein detoxification 21	Blue	
*GH_A08G2493*	*Gar08G28250*	Upstream activation factor subunit 30	Blue	
*GH_A08G2821*	*Gar08G31660*	Protein reversion‐to‐ethylene sensitivity 1	Turquoise	

Abbreviation: NA, no annotation; QTL, quantitative trait locus.

The leaf numbers, leaf lengths and widths, and seedling heights were measured and compared with those of TRV:*00* after 2 weeks by VIGS (Figure S7). For NB298, the sizes of the second and third true leaves were significantly bigger in TRV:*GH_A05G1367* than those of TRV:*00*. The sizes of the third true leaf and plant height were extremely significantly smaller or shorter in TRV:*GH_A06G2242* than those of TRV:*00*. The sizes of the third true leaf and plant height were significantly smaller or shorter in TRV:*GH_A08G2493* than that of TRV:*00*. For TM‐1, the sizes of the second true leaf were significantly smaller in TRV:*GH_A08G0993* and TRV:*GH_A08G2341* than those of TRV:*00*, and the plant heights were also significantly shorter than that of TRV:*00*, indicating that their growth and development of the third true leaf were inhibited by VIGS. The plant height was extremely significantly shorter in TRV:*GH_A08G1638* than that of TRV:*00*. Compared with TRV:*00*, polymerase chain reaction (PCR) results confirmed the expression levels of the corresponding genes in all VIGS plants were significantly reduced.

**FIGURE 4:**
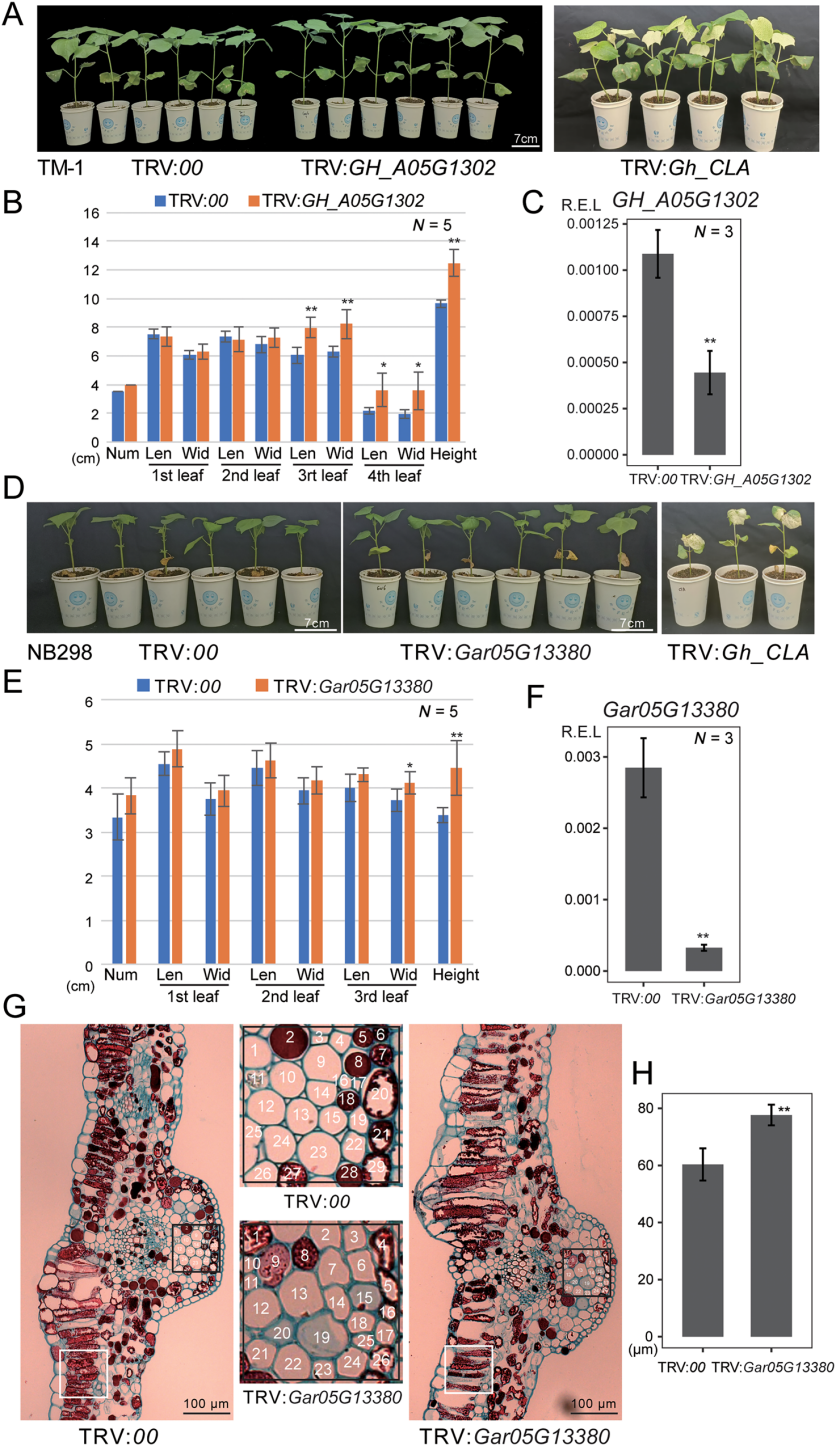
Phenotype after knockdown of *OFP17* (*GH_A05G1302/Gar05G13380*) gene at cotton seedling stage. (A) *GH_A05G1302*‐silenced seedlings by virus‐induced gene silencing (VIGS) in TM‐1, scale bar = 7 cm; (B) leaf sizes of *GH_A05G1302*‐silenced seedlings by VIGS in TM‐1; (C) the relative expression levels (R.E.L) of *GH_A05G1302* in TM‐1; (D) *Gar05G13380*‐silenced seedlings by VIGS in NB298, scale bar = 7 cm; (E) leaf sizes of *Gar05G13380*‐silenced seedlings by VIGS in NB298; (F) the R.E.L of *Gar05G13380* in NB298; (G) leaf cross‐section parts in TRV:*00* (left) and TRV: *Gar05G13380* (right), the magnified images in the black square frames with 100 µm (middle); and (H) the length of palisade cells in the white square frames with 100 µm. * and ** indicates the significance at the 0.05 and 0.01 levels, respectively.

Furthermore, NB298 and TM‐1 were also subjected to VIGS for knockdown of *Gar05G13380* and *GH_A05G1302* (homologous gene), respectively. *GH_A05G1302* (*GhOFP17*)‐silenced plants significantly increased leaf size and plant height in TM‐1 compared to the TRV:*00* (Figure 4A–C). Similarly, the width of the third true leaf in *Gar05G13380* (*GaOFP17*)‐silenced plants was bigger than that in the TRV:*00* plants (Figure 4D–F). Additionally, paraffin sections were performed on the quarters of the third true leaf of TRV:*00* and TRV:*Gar05G13380*. An area of 100 µm × 100 µm (black box) at the vein was selected to count the number of cells. We observed that there were 26 and 29 cells in TRV: *Gar05G13380* and TRV:*00*, respectively, implying that the cells of TRV:*Gar05G13380* plants could be larger than that of TRV:*00* plants. The palisade cells in TRV:*Gar05G13380* were also significantly longer than those in TRV:*00* (white box) (Figure 4G,H). Taken together, it is concluded that the expression level of the gene *OFP17* (where OFP is ovate family protein) may be negatively correlated with the cell size, thereby the high expression level resulting in small size of plant organs.

### 3.7 Functional analysis of putative genes by overexpression in *A. thaliana*

**FIGURE 5:**
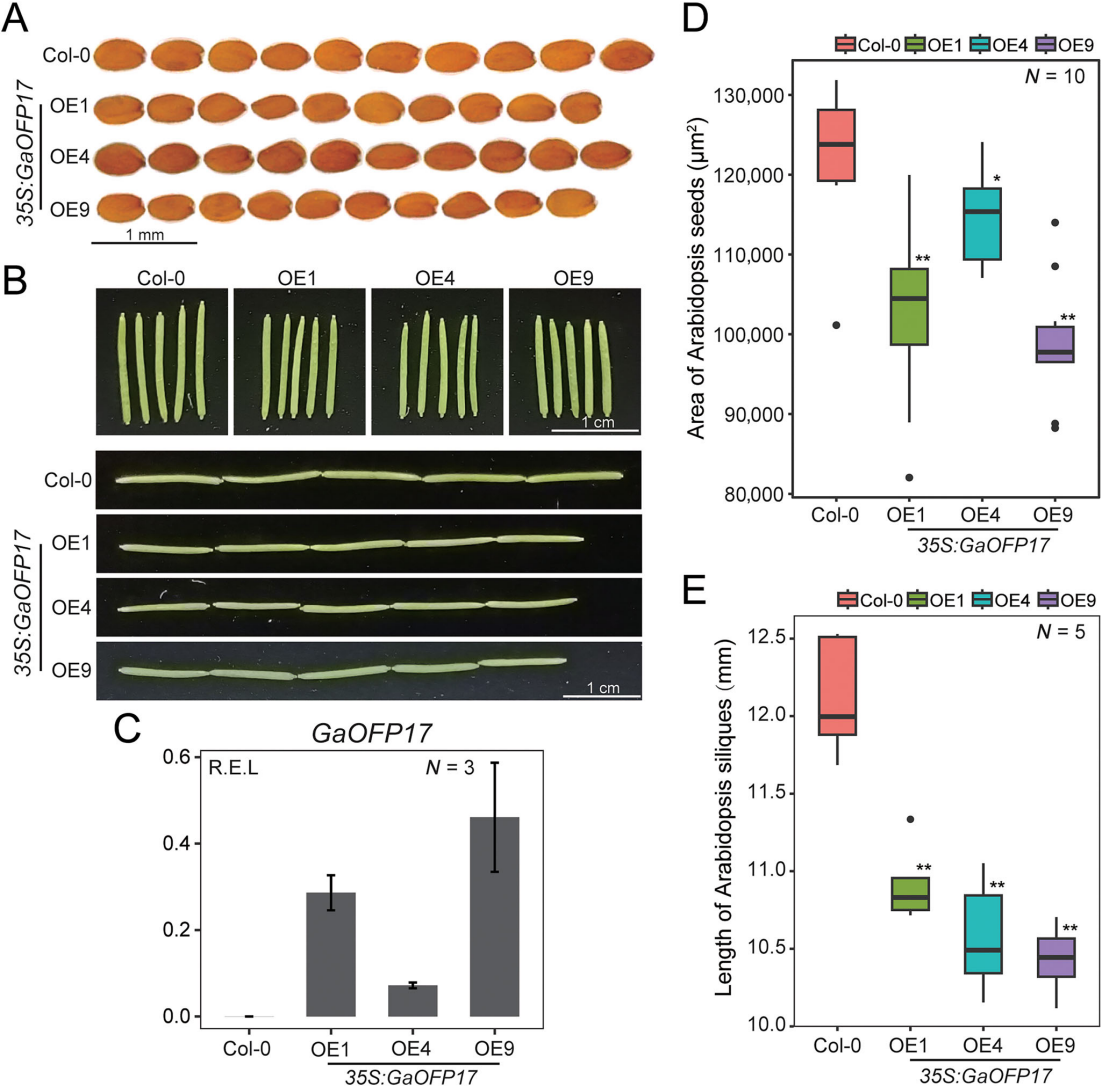
Effects of *GaOFP17* overexpression on sizes of seeds and siliques in *Arabidopsis thaliana*. (A) seeds, scale bar = 1 mm; (B) siliques, scale bar = 1 cm; (C) the relative expression levels (R.E.L) of *GaOFP17* in transgenic lines; (D) seed surface area; and (E) siliques length. * and ** indicates the significance at the 0.05 and 0.01 levels, respectively.* and ** indicates the significance at the 0.05 and 0.01 levels, respectively.

To further confirm the influence of these candidate genes on fruit/seed development, the full‐length CDS of the candidate genes were cloned into pBI121 to construct overexpression vectors and generate transgenic *A. thaliana* lines. Candidate genes (*Gar05G13380*, *Gar05G14070*, *Gar06G26100*, *Gar08G10510*, *Gar08G28180*, and *Gar08G28250*) with high expression levels in NB298 ovules (blue module) and candidate genes (*GH_A05G1324*, *GH_A06G1903*, *GH_A08G1638*, *GH_A08G2341*, and *GH_A08G2821*) with high expression levels in TM‐1 ovules (turquoise module) were transformed into *Arabidopsis* transformation, respectively. Three OEs for each gene were randomly selected to compare their seed sizes and fruits. Transgenic *Gar05G13380* (*GaOFP17*, homologous genes of *GH_A05G1302*) lines showed significantly smaller seed and fruit sizes than Col‐0 (Figure 5), while the overexpressed other gene lines showed no significant difference in seed size from Col‐0 (Figures S8 and S9).

Therefore, combining the results of the VIGS‐ed cotton seedlings and transgenic *A. thaliana* lines, it is concluded that the expression level of *OFP17* influences ovule growth and development, as well as regulates seed and fruit development.

## 4 DISCUSSION

### 4.1 Stable QTLs associated with cotton yield

Fruit is a plant organ specific to angiosperms that protects the ovule and seed during embryo development (Azzi et al., 2015). Several signaling pathways have been shown to control seed size by regulating the growth of maternal tissues, including or involving the ubiquitin‐proteasome pathway (Hao et al., 2021), G‐protein signaling (W. Yang et al., 2021), mitogen‐activated protein kinase signaling (L. Wang et al., 2021; M. Zhang et al., 2017), phytohormone perception and homeostasis (D. Liu, Zhao, et al., 2022), and some transcriptional regulators (Singh et al., 2021) in Arabidopsis and *O. sativa* (rice). Boll is the cotton fruit (capsule), and its size affects cotton yield and quality. Numerous QTLs/associated loci were identified for cotton BW and SI (Y. Hu et al., 2019; R. Liu et al., 2018; Zhu et al., 2021), but few of them have been cloned and characterized (X. Liu, Hou, et al., 2022). P. Wang et al. (2022) found that *GhUBC2L* modulates histone monoubiquitination synergistically with *GhUbox8* to regulate the expression of genes involved in organ development and cell cycle, thus controlling organ size in cotton. Cao et al. (2024) focused on *UDP‐glucosyltransferase 71C4* (*UGT71C4*) in cotton and found a member of the glycosyltransferase family that shapes seed width and length and influences SI and seed cotton yield.

*Gossypium arboreum* is one of the closest donor species of tetraploid cotton (Y. Hu et al., 2019). Here, NB298, a stable IL from *G. arboretum* into *G. hirsutum*, has significant differences from recurrent parent TM‐1 and contains introgressions of three chromosomes. Initially, we preliminarily mapped the QTL *q(BW+SI)‐A05* onto ChrA05 based on the F_2_ partial population. Near the *q(BW+SI)‐A05* interval, using the genome‐wide analysis study method, Feng et al. (2021) identified a BW‐related QTL, *qBW‐A05*, using the population of 289 *G. hirsutum–G. arboreum* ILs in four environments. Niu et al. (2023) identified a BW‐related QTL, *qBW‐C‐A05‐1*, using two *G. hirsutum* RIL populations. In this study, using newly developed markers, we finely mapped *q(BW+SI)‐A05* to the small interval of InDel‐4365–InDel‐4373 through the F_3_ and F_3:4_ populations of TM‐1 × NB298. Quantitative traits are often controlled by numerous QTLs, vice versa, the same QTL is also associated with two or more traits, and namely, the same QTL sometimes involves two or more traits, which is a multi‐QTL. Recently, several BW‐ and SI‐related multi‐QTLs have been identified in cotton. For example, Zhu et al. (2021) identified 95 candidate QTLs in more than two environments from 242 upland cotton accessions, among them 12 QTLs each for two traits and three QTLs each for three traits. Feng et al. (2021) found eight common‐QTL clusters with 38 QTLs on seven chromosomes.

### 4.2 Differential expression genes, except for *OFP17*, may not affect the sizes of tissues and organs in cotton

*GH_A08G2341* encodes the eukaryotic translation initiation factor 3 subunit A (eIF‐3), a large protein complex involved in most translation initiation processes, but little is known about the role of individual eIF‐3 subunits in plants. In all eukaryotes, *eIF‐3* functions to support the formation of translation initiation complexes and improve the accuracy of the start codon scanning mechanism (Raabe et al., 2019). The eIF‐3 complex specifically targets and initiates the translation of mRNAs involved in cell proliferation (Mayeur et al., 2003). Silencing of *OseIF3e* in rice showed the inhibited growth at the seedling and vegetative stages (W. Wang et al., 2016). In our study, *GH_A08G2341*‐silenced plant also showed the inhibited growth at the seedling stage, but *GH_A08G2341*‐overexpressed *Arabidopsis* had no significant difference from Col‐0 in seed size.

*GH_A05G1367* encodes the transmembrane 9 superfamily member 7 (TM9SF), which may function as an effector for cellular copper homeostasis (https://www.uniprot.org/). Structurally, all *TM9SF* contain a large non‐cytoplasmic region (a variable extracellular N‐terminal domain) and nine transmembrane domains, and they are highly conserved during evolution (Froquet et al., 2008). Here, we found that silencing *GH_A05G1367* gene significantly increased the size of leaves in cotton seedling; however, there was no significant difference in seed size between *GH_A05G1367*‐overexpressed *Arabidopsis* lines and Col‐0.

The expression level of the *GH_A06G2242* in NB298 was higher than that in TM‐1; when silencing the *GH_A06G2242* gene, the leaf sizes of cotton seedling were significantly reduced, and the traits of TRV:*GH_A06G2242* were inconsistent with the level of gene expression. Particularly, there was no significant difference in seed size between the transgenic *GH_A06G2242* lines and Col‐0. *GH_A06G2242* encodes the pyruvate dehydrogenase E1 component subunit beta‐2 (PDH‐E1). Pyruvate dehydrogenase is an enzyme (E1) of the PDH complex (PDC). This multi‐enzyme complex includes pyruvate dehydrogenase (E1), dihydrolipoyl transacetylase (E2), and dihydrolipoyl dehydrogenase (E3). It controls the entry of carbon into the mitochondrial tricarboxylic acid cycle to produce energy for cells. The E1 component of PDC consists of an E1α catalytic subunit and an E1β regulatory subunit. In *A. thaliana*, mitochondrial PDC E1 contributes to auxin transport during tissue development (Ohbayashi et al., 2019).

*Gh_A05G1302* encodes the *OFP17* (https://cottonfgd.org), which can regulate multiple aspects of plant growth and development (https://www.uniprot.org/). OFPs are a new type of transcription factor family, which were first discovered in the study of *Solanum lycopersicum* (tomato). So far, *OFPs* have been identified through the conserved OVATE domain in plants (Dangwal & Das, 2018). Overexpression of *OFPs* usually results in plants with relatively shorter and thicker tissues, such as kidney‐shaped cotyledons or rounder leaves and fruits. Overexpression of *OVATE* and *SlOFP20* in tomato leads to small floral organs and leaves, a phenotype being similar to that caused by overexpression of *AtOFP1* (Wu et al., 2018). Likewise, overexpression of several rice *OFPs* exhibited the same results, with the reductions in plant height, spikelet size, seed width, and leaf width. The interaction of AtOFP with TALE proteins (KNOX and BELL) (Hackbusch et al., 2005) can regulate secondary cell wall biosynthesis (Y. Liu & Douglas, 2015), ovule development (Pagnussat et al., 2007), and the timing of the transition from the vegetative stage to the reproductive stage (L. Zhang et al., 2016). Additionally, OsOFPs in rice and GhOFP4 in cotton also interact with TALE proteins, play roles in secondary cell wall biosynthesis, and participate in vascular bundle localization and hormone responses in rice (Gong et al., 2014; Schmitz et al., 2015; C. Yang et al., 2018). This suggests that *OFPs* play a role in regulating tissue shape during the development of various plants (Schmitz et al., 2015; Xiao et al., 2017; C. Yang et al., 2018, 2016), even though future CRISPR‐based or transgenic validation would be necessary.

## AUTHOR CONTRIBUTIONS

**Min Xu**: Conceptualization; data curation; methodology; writing—original draft. **Liuchun Feng**: Conceptualization; methodology. **Susu Liu**: Formal analysis. **Zhechang Zhang**: Formal analysis. **Chenhui Zhou**: Data curation. **Guoli Feng**: Data curation. **Ningshan Wang**: Data curation. **Nijiang Ai**: Data curation. **Baoliang Zhou**: Conceptualization; funding acquisition; project administration; resources; supervision; writing—review and editing.

## CONFLICT OF INTEREST STATEMENT

The authors declare no conflicts of interest.

## Supporting information



Figure S1 Collinearity analysis of the A_2_ and A_t_ genomes.
Figure S2 Frequency distributions of BW, SI and BN in the F_2_ and F_3_ populations.
Figure S3 QTLs for yield‐related traits in the F_2_ and F_3_ populations.
Figure S4 Principal component analysis (PCA) plots and differential gene profile.
Figure S5 WGCNA of DEGs from ovules during the time courses of the developmental stage in TM‐1 and NB298.
Figure S6 Gene modules related to ovule development (blue, turquoise).
Figure S7 Embryo‐related genes silenced by VIGS in cotton at seedling stage.
Figure S8 Seeds and their sizes in purified transgenic Arabidopsis lines in box plot.
Figure S9 *GaOFP17*‐overexpressed *Arabidopsis thaliana* morphological traits at different developmental stages.

Table S1 Primers used to clone gene fragment and full‐CDS in this study.
Table S2 Primers used for RT‐qPCR in this study.
Table S3 Boll weight, seed index and boll number between TM‐1 and NB298 in three environments.
Table S4 Primers used for QTL mapping.
Table S5 Basic parameters of yield‐related traits in the F_2_ and F_3_ populations.
Table S6 Correlation coefficients between yield‐related traits in the F_2_ and F_3_ populations.
Table S7 QTL localization of yield‐related traits in the F_2_ population.
Table S8 QTL fine mapping of three yield‐related traits in the F_2_ and F_3_ populations.
Table S9 The introgression and BW and SI value of recombinants and parents.
Table S10 Quality of reads for 18 RNA‐aeq samples

## Data Availability

The data that support the findings of this study are available from the corresponding author upon reasonable request. The cotton gene sequences used in this study are downloaded from Cotton database (http://cotton.zju.edu.cn). The accession numbers of the genes are as follows: *GhOFP17* (GH_A05G1302), *GhCOX19* (GH_A05G1324), *GhTMN7* (GH_A05G1367), NA (GH_A06G1903), *GhPDH‐E1* (GH_A06G2242), *GhRPS11* (GH_A08G0993), *GhGATA4* (GH_A08G1638), *GhTIF3A1* (GH_A08G2341), *GhDTX21* (GH_A08G2485), *GhUAF30* (GH_A08G2493) and *GhRTE1* (GH_A08G2821) (https://cottonfgd.org).
