# Supplementary material for: Fine mapping and functional analysis of the candidate gene OFP17, regulating both seed and fruit sizes in cotton
Source: Plant Genome. 2026 Jan 31;19(1):e70195. doi: 10.1002/tpg2.70195 (PMC12871568; doi:10.1002/tpg2.70195)
Supplement: Supplementary file 1 [file TPG2-19-e70195-s002.docx]

**Figure S1: Collinearity analysis of the A_2_ and A_t_ genomes.** (A) Collinearity analysis among all 13 chromosomes; (B) Collinearity analysis of Chr08 chromosome. Genic synteny blocks are connected by gray lines, with green indicating collinearity on the introgression fragments.

**Figure S2: Frequency distributions of BW, SI and BN in the F_2_ and F_3_ populations.**

**Figure S3: QTLs for yield-related traits in the F_2_ and F_3_ populations.** (A) Genetic linkage maps of the introgression segments on Chrs. A05, A06 and A08; (B) QTL mapping for BW and SI in the F_2_ populations; (C) Genetic linkage map on Chr. A05 constructed by newly developed markers in the *q(BW+SI)-A05* region; (D) QTL mapping of BW and SI in the F_2_ populations of *q(BW+SI)-A05* region; (E) QTL mapping of BW, SI and BN in the F_3_ populations of *q(BW+SI)-A05* region.

**Figure S4: Principal component analysis (PCA) plots and differential gene profile. (A)** PCA plots of RNA-seq samples**.** (B) Comparison and UpSet plot of ovules at different times in TM-1 and NB298. (C) Venn diagram of differential genes of NB298 and TM-1 in ovules at 0, 3 and 5 dpa.

**Figure S5: WGCNA of DEGs from ovules during the time courses of the developmental stage in TM-1 and NB298.** (A) Network topology of different soft-thresholding power. The left shows the scale-free fit index corresponding to various soft-thresholding power (β). The right shows the average connectivity corresponding to various soft-thresholding power; (B) Determination of soft-thresholding power in WGCNA. The left figure shows the histogram of connectivity at β=7. The right denotes the detection of scale-free topology for β=7; (C) Hierarchical clustering showing co-expressed gene modules identified by WGCNA. Dissimilarity based on topological overlap matrices (1-TOM); (D) The obtained co-expression modules and the number of the genes in corresponding modules; (E) Heatmap of the correlation between modules and sample traits, correlation values and *P* values in parentheses are included within each box.

**Figure S6: Gene modules related to ovule development (blue, turquoise).** (A) Correlation of traits with module characteristic vector genes; (B) Gene expression levels of the target module (blue, turquoise); (C) The values of the target module eigengenes in different samples (blue, turquoise); (D) Scatterplots of correlation between module membership (MM) and gene significance (blue, turquoise); (E) Scatterplots of correlation between intramodular connectivity and gene significance (blue, turquoise); (F) Visualization of co-expression networks by Cytoscape software, each module display top 50 genes, solid circles represent gene from introgression fragments, hollow circles denote genes that did not undergo introgression. Interaction network node size is proportional to the degree of the node. The gene names are shown for the selected hub genes of interest.

**Figure S7: Embryo-related genes silenced by VIGS at seedling stage in cotton.** (A, D, G, I, L, O) Cotton after knockdown the candidate genes at seedling stage; (B, C, E, F, H, J, K, M, N, P) The left figure shows the plant height and leaf sizes of cotton seedlings after knockdown the candidate genes; the right figure corresponds to the relative expression level (R.E.L) of candidate genes; TRV:*00* as the negative control and TRV:*Gh_CLA* as the positive control.

**Figure S8: Seeds and their sizes in purified transgenic Arabidopsis lines in box plot.** (A, B, D) Seeds of transgenic Arabidopsis; (C, E) Seed sizes of transgenic Arabidopsis.

**Figure S9: *GaOFP17*-overexpressed *Arabidopsis thaliana* morphological traits at different developmental stages.** (A) Seedlings; (B) Bolting.
